# Efficacy of Topical Treatment of Low-Risk Cervical Preinvasive Lesions with Glycyrrhizinic Acid

**DOI:** 10.3390/jcm14010136

**Published:** 2024-12-29

**Authors:** Diana Andzane, Jana Zodzika, Irina Jermakova, Ilva Senfelde, Marina Utorova, Dace Rezeberga

**Affiliations:** 1 Gynaecology Clinic, Riga East Clinical University Hospital, Hipokrata Street 2, LV 1079 Riga, Latvia; jana.zodzika@rsu.lv (J.Z.); irina@qprakse.lv (I.J.); ilva.senfelde@inbox.lv (I.S.); marina.utorova@aslimnica.lv (M.U.); dace.rezeberga@rsu.lv (D.R.); 2 Department of Obstetrics and Gynaecology, Rīga Stradiņš University, Miera street 45, LV 1013 Riga, Latvia; 3 Riga Maternity Hospital, Miera Street 45, LV 1013 Riga, Latvia; 4 Department of Clinical Skills and Medical Technologies, Rīga Stradiņš University, Anninmuizas bulvaris 26a, LV 1067 Riga, Latvia

**Keywords:** cervical intraepithelial neoplasia, human papillomavirus, glycyrrhizinic acid, colposcopy, cervical precancerous disease

## Abstract

**Background/Objectives**: The study aimed to investigate the efficacy of medication treatment with glycyrrhizinic acid for cervical intraepithelial neoplasia (CIN) 1 lesions. **Methods**: Women with histologically confirmed CIN 1 in cervical biopsies were included in the prospective study. Participants of the study group used glycyrrhizinic acid spray (Epigen spray) topically 10 days (Epigen 10-day subgroup) or 20 days (Epigen 20-day subgroup) per month for 6 months. Women in the control group had no treatment. There were two follow-up visits 6 months apart. All patients were screened for human papillomavirus (HPV) before enrollment and during the first follow-up visit. **Results**: There were 50 patients in the Epigen group and 50 patients in the control group. At the first follow-up visit, in the histological findings, progression to CIN 2+ was 6.7% in the Epigen 20-day subgroup, 31.1% in the control group, and the persistence of CIN 1 was 86.7% in the Epigen 20-day subgroup and 62.2% in the control group, *p* = 0.03. Large loop excision of the transformation zone (LLETZ) was statistically significantly more frequent in the control group after the first follow-up visit (10% in the Epigen 20-day subgroup vs. 30% in the control group, *p* = 0.032). No such relationships were found during the second follow-up visit. **Conclusions**: The vaginal spray of the glycyrrhizinic acid used 20 days per month appears to decrease the risk of the progression of CIN 1 lesions, with the need to perform LLETZ. However, a similar effect is not observed after stopping usage.

## 1. Introduction

The most common cervical precancerous disease is low-risk cervical intraepithelial neoplasia or CIN 1. The lesion spontaneously regresses in 60%, persists in 30%, and progresses to CIN 3 in 10% and to invasion in 1% of cases [1]. If there is a biopsy-proven histological low-risk squamous intraepithelial lesion (LSIL), a repeat cervical test (cytology +/ high-risk human papillomavirus (HR HPV) test) should be performed in 12 months. Usually, treatment of CIN 1 should be avoided [2]. Although most LSIL lesions will regress, around 1.4% will persist for many years [3], and managing the long-term persistent low-grade lesion is unclear. At some point, treatment with cervical excision or ablation might be considered. Studies have shown that cervical excision is associated with perioperative complications and adverse pregnancy outcomes like preterm labor. Although ablative techniques have less harmful effects on women’s health [4,5], there is a growing interest in the non-invasive management of CIN 1. Evidence of effective medical treatment for HPV infection and related cervical lesions is missing.

Studies have shown that metabolites isolated from licorice (Glycyrrhiza glabra), such as glycyrrhizinic acid, have many pharmacological activities, such as anti-inflammatory, antiviral, participation in immune regulation, anti-tumor, hepatoprotective, and other activities [6,7]. Glycyrrhizinic acid as a sweetener is currently considered “generally recognized as safe” by the US Food and Drug Administration [6]. Licorice and glycyrrhizin-related side effects are hypokalemia and hypertension in the case of peroral herbal drug or tea chronic use [8,9].

Although there are some data showing that the topical application of glycyrrhizinic acid to the cervix in patients with HPV infection might improve the protective properties of the cervical mucosa by reducing pro-inflammatory and increasing anti-inflammatory cytokine concentrations [10], it is not clear if medical treatment of CIN 1 with glycyrrhizic acid could reduce persistent HPV infection, the need for prolonged follow-up CIN 1 cases, and the use of more aggressive treatment methods. This study aimed to investigate the efficacy of medication treatment for CIN 1 lesions. We hypothesized that the local application of glycyrrhizinic acid to the cervix promotes HPV clearance and reduces the progression of CIN 1 to high-grade lesions.

## 2. Materials and Methods

### 2.1. Design

An open-label randomized study was conducted at the Riga East University Hospital from July 2017 to May 2022. Women with abnormal cervical cytological screening test results were referred to the colposcopy unit of the hospital for colposcopic evaluation. The indication for colposcopy was abnormal results of cervical cytological screening test: high-risk squamous intraepithelial lesion (HSIL), two consequent LSIL test results six months apart, or three consequent atypical squamous cells of undetermined significance (ASCUS) test results six months apart. Until July 2022, no primary HPV screening had been introduced in Latvia. Colposcopies were performed by a certificated colposcopy specialist (Jana Zodzika). In the case of visual suspicion of cervical pathology, at least two biopsies were taken. A histological examination was performed at the Pathology Center of Riga East University Hospital.

Women with the first-time histologically confirmed CIN 1 in cervical biopsies were contacted by telephone (as previously agreed), informed about biopsy results, and invited to participate in the study. The inclusion criterion was histologically confirmed CIN 1 in cervical biopsies. The exclusion criteria were the patient’s refusal to participate in the study, pregnancy, or previous history of cervical precancerous abnormalities, including any cervical treatment.

There were three study visits—the enrollment and allocation and the first and second follow-up visits. During the enrollment and allocation visit, all patients signed an informed consent form. The physician filled out a questionnaire about the patient’s general state of health, medical history, sexual health, and contraception. Per speculum examination was conducted, and a cervical smear was taken for further HPV testing. Women were randomly included in the study (Epigen group) and control group. For allocation, women who underwent colposcopy on even dates were invited to the control group, but those who had colposcopy on odd dates were included in the Epigen group. If patients who were assigned to the intervention group disagreed with the use of medication but agreed to have follow-up and participate in the study, they were included in the control group. Participants of the study group members used glycyrrhizinic acid spray (Epigen^®^ spray, Chemigroup France, S.A., Chatou, France; Cheminova Internacional, S.A., Madrid, Spain) topically twice daily for six months. Women who were enrolled in the study during the first study year used glycyrrhizinic acid spray 10 days per month (the Epigen 10-day subgroup); after revision of the preliminary study results, it was decided to use glycyrrhizinic acid spray 20 days per month (the Epigen 20-day subgroup) during the rest of the study period. Women in the control group had no treatment. The patients in the Epigen group filled out a diary in which they noted the times of use of the preparation and any side effects.

During the first follow-up visit, 6 months after the allocation, smears for cytological and HPV testing were taken, and colposcopic and histological examinations were conducted. If CIN 2/3 was detected at the first follow-up visit, women underwent large loop excision of the transformation zone (LLETZ) and were no longer invited for a second visit. Their histological changes were assessed as deterioration in the first follow-up visit analysis, and they were not included in the analysis for the second follow-up visit.

During the second follow-up visit, 6 months after the first one, smears for cytological testing were taken, and colposcopic and histological examinations were conducted. Cervical biopsies at the follow-up visits were not performed on patients who had become pregnant. In this case, they were not included in the analysis of the histological results of the respective follow-up visit but continued to participate in the analysis of cytological and colposcopic changes.

HPV genotyping, including HPV 6, 11, 16, 18, 26, 31, 33, 35, 39, 44, 45, 51, 52, 53, 56, 58, 59, 66, 68, 73, and 82 types, was conducted by real-time–polymerase chain reaction (PCR). For quantitative detection of HPV-type deoxyribonucleic acid (DNA), the Sacace (Italy) PCR kit was used, while the Seegene (South Korea) kit was applied for qualitative HPV detection. The Joint Laboratory of Clinical Immunology and Immunogenetics, Rīga Stradiņš University, performed HPV tests.

Before the beginning of the study, permission was obtained from the Rīga Stradiņš University Research Ethics Committee (Nr. 12/25.05.2017).

### 2.2. Interpretation of Results

An improvement of the examination was considered if cytological results were ASCUS or negative for intraepithelial lesions and CIN lesions were no longer detected at histological analysis; changes were considered constant if CIN 1 was detected histologically in at least one biopsy sample and were considered as deterioration if CIN 1 progressed to CIN 2+ in at least one biopsy sample.

A new high-risk HPV infection was defined as the appearance of a new virus type in a woman at the first follow-up visit that was not detected by HPV testing at the time of the patient’s inclusion in the study. High-risk HPV clearance was defined if, at the first follow-up visit, the patient was negative for all types of high-risk HPV and had no new high-risk HPV infection (high-risk HPV-negative).

### 2.3. Methods of Statistical Analysis

The data obtained were processed with MS Excel Version 2111 and IBM SPSS Statistics 22.0 software.

Chi-square and Fisher’s exact test were used for the statistical analysis of nonparametric data. Independent two-sample Student’s *t*-test was used to analyze two groups of parametric data; for the analysis of more than two groups, analysis of variance (ANOVA) was used. The result was considered statistically significant if the confidence level was *p* < 0.05. Intention-to-treat analysis was performed for all randomized patients, and per-protocol analysis was performed for patients who came to all indicated follow-up visits and completed the study.

## 3. Results

**Figure 1:**
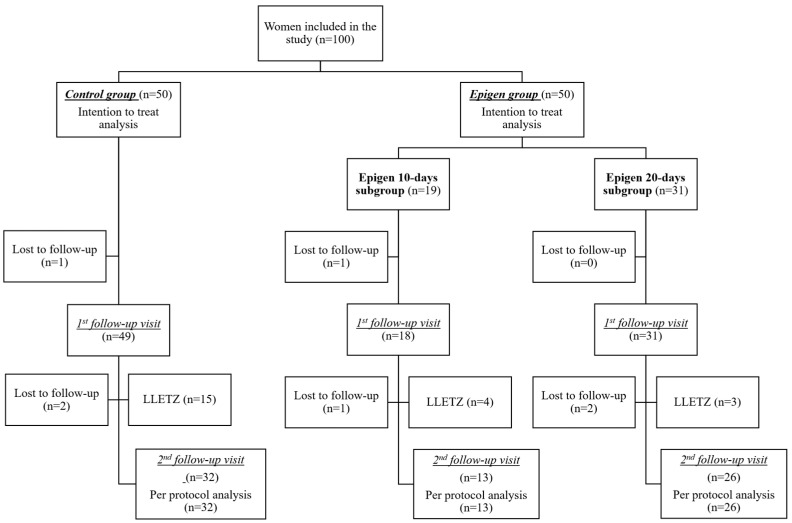
Flowchart of the study population. LLETZ—large loop excision of the transformation zone.

There were 100 patients with CIN 1 included in the study: 50 women in the Epigen group and 50 women in the control. In the Epigen 10-day subgroup,19 took part, but in the Epigen 20-day subgroup, 31 women were involved. An intention-to-treat analysis was performed for this population. At the first follow-up visit, two women were lost to follow-up: one in the control and one in the Epigen 10-day subgroup. At the second follow-up visit, five women were lost in total. Per-protocol analysis was performed in the population of women who completed the study: 32 women in the control and 39 women in the Epigen group (see Figure 1). Five patients refused to participate in the study and were not included in the study.

At the first follow-up visit, six patients were pregnant: one in the Epigen 20-day subgroup, one in the Epigen 10-day subgroup, and four in the control group. At the second follow-up visit, there were two other pregnant patients: one in the Epigen 10-day subgroup and one in the control group. Cervical biopsy was not performed on these pregnant patients; however, for all other patients who attended the follow-up visits, cytology, colposcopy, and biopsy were performed.

**Figure 2:**
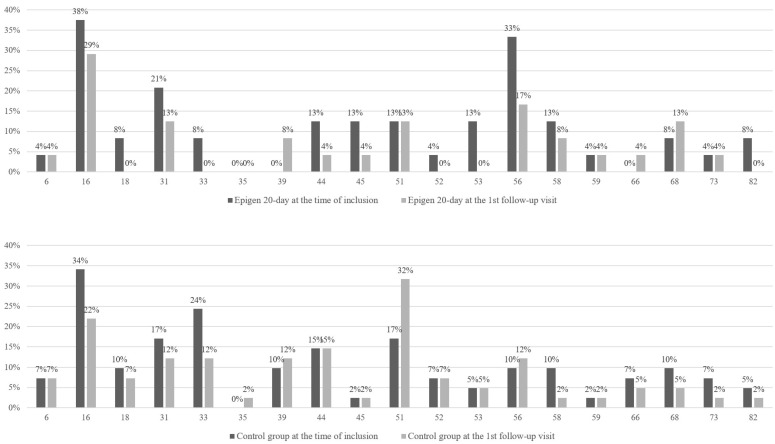
Comparison of the type-specific human papillomavirus (HPV) incidence in the Epigen 20-day subgroup and control group at the inclusion and the first follow-up visit. Low-risk HPV types: 6; 44; 73; 82. HPV 53—probable high-risk type.

There was no statistically significant difference between the Epigen and the control group in background parameters and HR HPV prevalence at the inclusion, but in the Epigen group, there were more women with higher education (Table 1). HPV types 16, 31, 33, 51, and 56 were the most often detected among enrolled women. There was no statistically significant difference in distribution and changes in virus concentration over time between groups (Figure 2).

Medication was used in 20 out of 50 participants according to the recommended protocol. They used the medication for at least 95% of the proposed applications (at least 228 times out of 240 applications in the Epigen 20-day subgroup and 114 out of 120 applications in the Epigen 10-day subgroup). The remaining 30 patients applied the medication for at least 75% of the required applications (at least 180 out of 240 applications in the Epigen 20-day group and 90 out of 120 applications in the Epigen 10-day group). Three women noted a burning sensation after taking the medication for a few days. No serious treatment-associated side effects were observed.

### 3.1. Comparison Between Epigen 20-Day and Control Group

**Figure 3:**
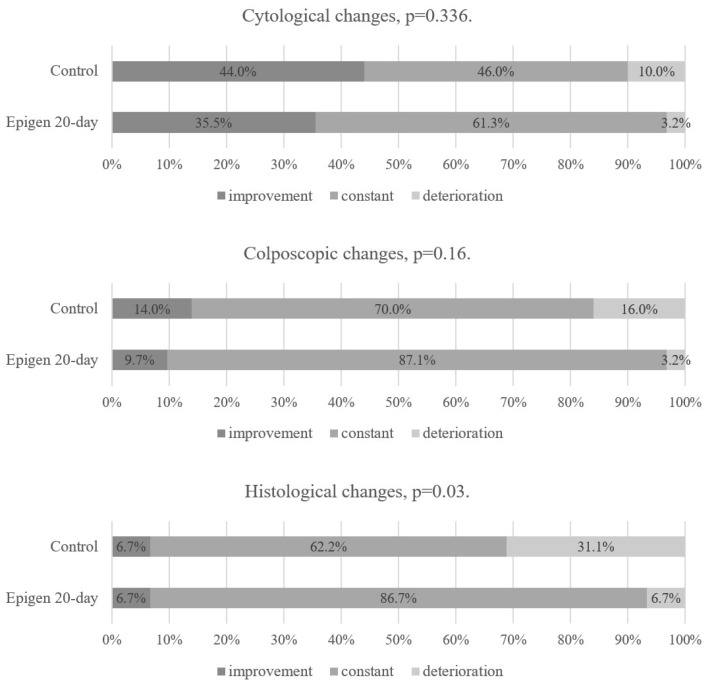
Epigen 20-day and control group results at the 1st follow-up visit. Intention-to-treat analysis.

When comparing the Epigen 20-day subgroup to the control group, there were no statistically significant differences in cytological and colposcopic changes at the first follow-up visit. However, in histological findings, progression to CIN 2+ was more common in the control group (7% in Epigen 20-day and 31% in the control group), improvement was similar in both groups (7%), and the persistence of CIN 1 was more common in the Epigen 20-day subgroup (87%) than in the control group (62%) (Figure 3). LLETZ after first follow-up visit was also statistically significantly more frequent in the control group (9.7% in the Epigen 20-day subgroup vs. 30.6% in the control group, *p* = 0.032). In patients who underwent LLETZ, the histological findings of excisional material included the following lesions: CIN 1—one case, CIN 2—two cases in the Epigen 20-day subgroup, and CIN 1—two cases, CIN 2—eight cases, and CIN 2/3–five cases in the control group.

**Figure 4:**
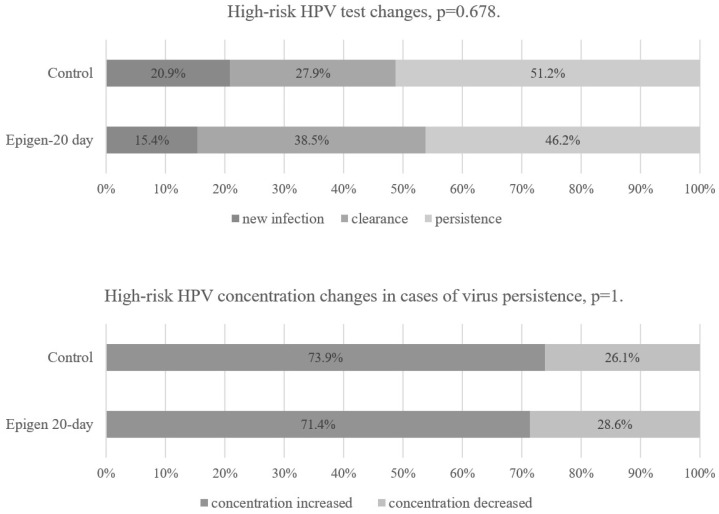
High-risk human papillomavirus (HPV) changes after the 1st follow-up visit.

Clearance, new infection, or persistence of HPV was equally common in the Epigen 20-day subgroup and control group (Figure 4). During the first follow-up visit, the incidence of HPV 16/18 was not different between the control and Epigen 20-day subgroup (13 out of 49 versus 7 out of 31, *p* = 0.451). However, in the control group, 10 out of 15 (66.7%) women who underwent LLETZ had HPV types 16/18 positive compared to 3 out of 34 (8.8%) women who did not have LLETZ, *p* < 0.001. During the first follow-up visit in the Epigen 20-day subgroup, two out of three patients who underwent LLETZ tested positive for HPV types 16/18, while among those who did not have LLETZ, five out of 28 (17.9%) tested positive, *p* = 0.12.

**Figure 5:**
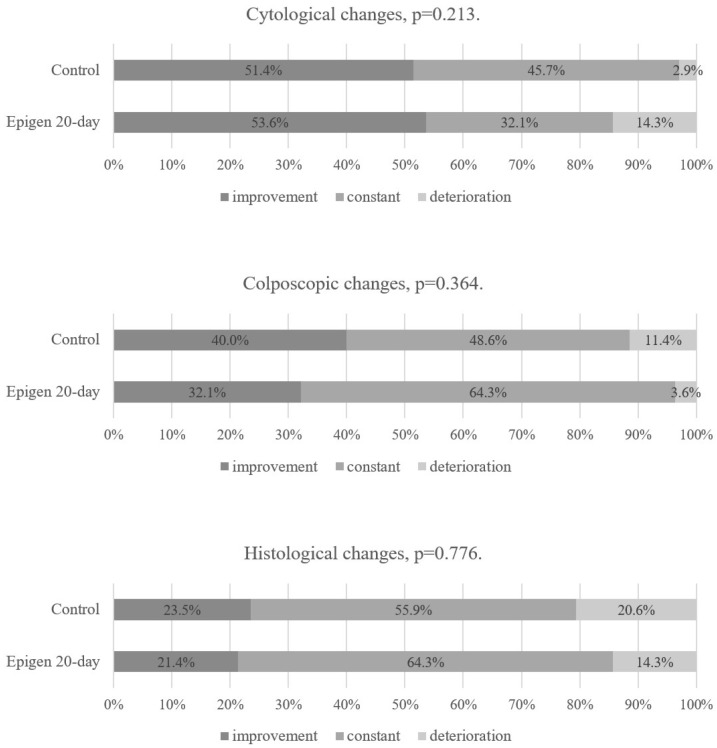
Epigen 20-day and control group results at the 2nd follow-up visit. Intention-to-treat analysis.

**Figure 6:**
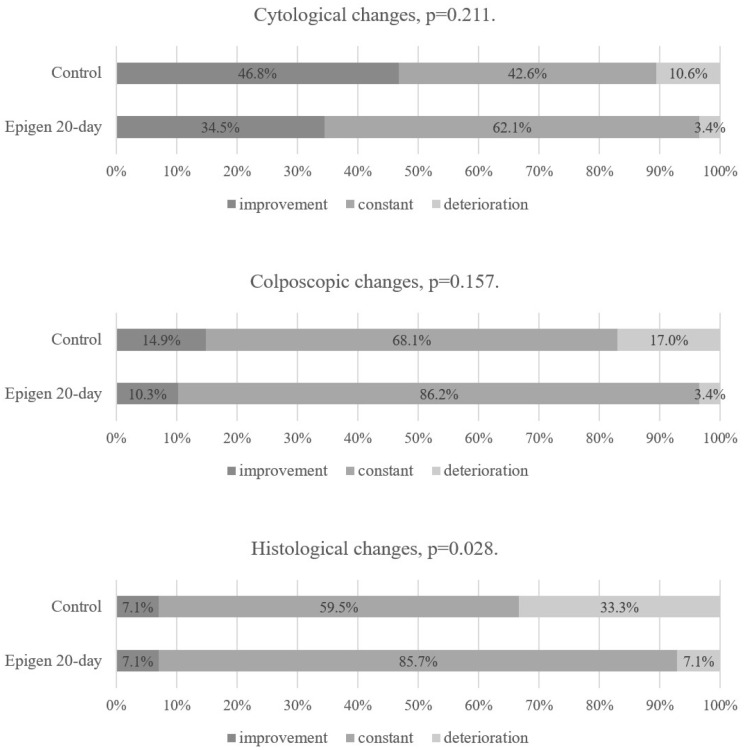
Epigen 20-day and control group results at the 1st follow-up visit. Per-protocol analysis.

**Figure 7:**
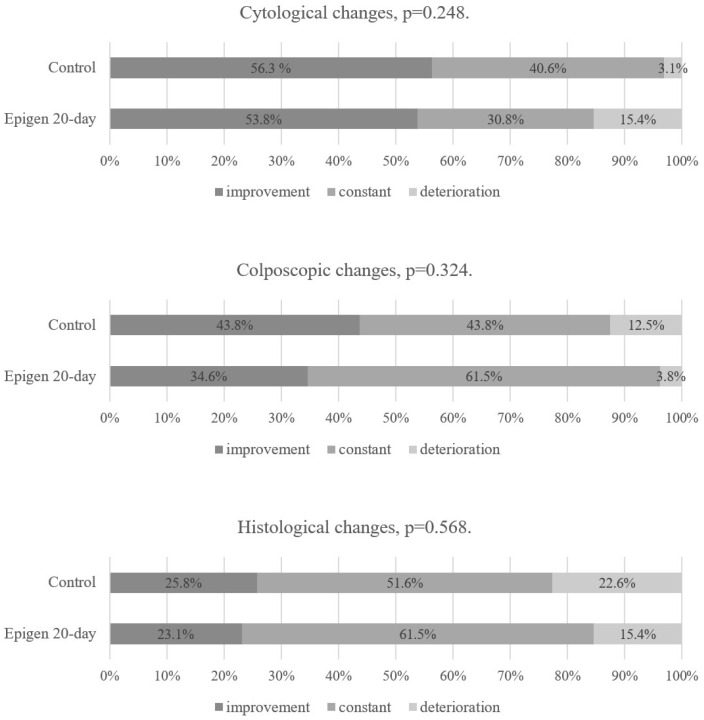
Epigen 20-day and control group results at the 2nd follow-up visit. Per-protocol analysis.

There was no statistical difference between the Epigen 20-day subgroup and the control group in terms of cytological, colposcopic, or histological changes at the second follow-up visit in the intention-to-treat analysis (Figure 5). The same results were found in the per-protocol analysis as in the intention-to-treat analysis when comparing Epigen 20-day with the control group at the first and second follow-up visits (Figure 6 and Figure 7). Since all patients applied the medication at least 75% of the required times, none were excluded from the per-protocol analysis.

### 3.2. Comparison Between Epigen 10-Day and Control Group

No statistically significant difference was found in the incidence of improvement or deterioration in cytological, colposcopic, and histological findings between Epigen 10-day use and the control group at either the first or second follow-up visit (Table 2). In the Epigen 10-day subgroup, LLETZ was required for four patients after the first follow-up visit. In these patients, the histological findings of the excisional material included the following lesions: CIN 2—one case, CIN 2/3—three cases.

### 3.3. Comparison Between Epigen and Control Group

A statistically significant difference was not found when comparing the Epigen group (in total) and the control group.

## 4. Discussion

In the current study, we found that women with low-grade cervical precancerous disease who used Epigen vaginally 20 days per month for six months had less common progression of the lesion within six months.

If CIN 1 progresses into CIN 2+, LLETZ is indicated. This procedure is associated with perioperative complications and adverse pregnancy outcomes [5]. Therefore, different pharmacological methods for the treatment of CIN are currently being actively studied, such as Imiquimod, 5-Fluorouracil, retinoic acid, Cidofovir, trichloroacetic acid, and Coriolus versicolor-based vaginal gel [11,12,13,14]. Imiquimod shows the most promise in the immune modulators group [13]. A nonrandomized multicenter trial (TOPIC-3) provides evidence for topical imiquimod cream as a feasible and safe treatment for high-grade CIN [15]. High-grade dysplasia regression occurred in 60% of women after treatment with imiquimod and 95% after LLETZ, and high-risk HPV clearance occurred in 69% and 67% in the imiquimod and LLETZ groups, respectively. Although the effectiveness is considerably lower than LLETZ treatment, authors note that imiquimod treatment could prevent initial surgical treatment in over 40% of women and should be offered to a selected population of women who wish to avoid (repeated) surgical treatment of high-grade CIN [15]. Other multicenter, randomized controlled trials show that in women with HSIL, imiquimod treatment results in lower HPV clearance rates when compared to LLETZ (43% in the Imiquimod group versus 64% in the LLETZ group), with histologic regression of 63% and complete histologic remission of 37% [16]. In the Rahangdale et al. study, 5-Fluorouracil resulted in 93% regression of CIN 2 versus 56% for placebo [17]. The metanalysis of the five retinoids has not found them to be effective [13]. Of the antivirals, cidofovir has demonstrated promising results [13]. In the study by Van Pachterbeke et al., the histologic remission of any CIN was demonstrated in 61% compared with 20% in the placebo group for CIN 2 [18]. In the prospective, nonrandomized, single-arm study (TRICIN) topical treatment with trichloroacetic acid-caused histologic complete remission of CIN 1 and CIN 2 lesions in 78.4% of cases [14]. The efficacy of Coriolus versicolor-based vaginal gel was studied in the multicenter, open-label, randomized, parallel-group study. HR HPV clearance occurred in 63% of treated patients and 40% of untreated patients [12]. The current study showed that the vaginal spray of glycyrrhizinic acid used for 20 consecutive days followed by seven days with no treatment regime decreased the risk of the deterioration of CIN 1 lesions and the frequency of LLETZ performing. Still, a similar effect was not observed after stopping the usage. The indication for performing LLETZ was a histologically confirmed CIN 2+ lesion in the biopsy. However, in some patients from both the Epigen and control groups, the LLETZ histological material revealed only CIN 1 lesions. For these patients, the histological assessment was considered a progression because the definitive histology is the highest grade from any diagnostic or therapeutic biopsies [19]. It is possible that the CIN 2+ lesion was entirely removed during the biopsy or that clearance of the lesion happened due to a biopsy-induced immunological reaction [20]. Although in the present study, the glycyrrhizinic acid used locally decreased the risk of the deterioration of CIN 1 lesions, we were not able to show HPV clearance or decrease in concentration or prevention from the new infection. The literature describes that CIN 1 progression to the high-grade abnormalities requiring LLETZ occurs in up to 20% of cases [1]. However, in our study, the rate was 30% in the control group. This can probably be explained by the more common HPV 16/18 types of infection in the control group among those with disease progression, while this was not observed in the Epigen 20-day subgroup. Glycyrrhizinic acid acts on pathogenic factors by immunomodulatory, antiviral, and anti-inflammatory action [7]. It is possible that Epigen influences immunological mechanisms that may provide protection against the effects of HPV. The lack of a stable effect of glycyrrhizinic acid on CIN 1 could be explained by the fact that it does not affect the elimination of the HPV from the cervix, which is the etiological factor of the lesion. When the use of the preparation is stopped, the inflammatory reaction may return, and cervical dysplasia progresses. Nevertheless, further studies are needed to confirm this hypothesis.

A study by Valencia et al. also evaluated the effectiveness of glycyrrhizinic acid on genital infections due to HPV and LSIL [21]. In their study, LSIL was present in 40% of 62 patients. A monthly Pap smear and colposcopy were used for control. As a result, LSIL persisted in 27.7% of cases and only one progressed to CIN 2. In our study, in the Epigen 20-day subgroup, cytological persistence of LSIL occurred in 61.3% and the persistence of colposcopic changes in 87.1% at the first follow-up visit. The significant difference in the results can be explained by the small number of participants with LSIL in the Valencia et al. study and the combined use of oral and topical preparation [21].

Different factors may impact the natural history of HPV infection and LSIL—like age, smoking status, HPV type, etc. All baseline parameters except education were comparable between treatment and control groups in the current study. There were more women with higher education in the Epigen group, probably due to randomization principles: if a woman did not agree to take medicament, she was included in the control group. More educated women understand better the meaning of the trials in medicine, but this should not affect the results of the study.

In general, the parameters of our study population at baseline are comparable to other similar studies. The average age of patients in this study was 32 years. In other studies, the average age of patients with CIN 1 varied from 27 to 40 years [3,12,14,21]. Additionally, 38% of participants smoked in the TRICIN study [14], 26% in the Ciavattini et al. study [3], and 30% in our study. In our study population, HPV type 16 was the most common (36% at inclusion among patients with identified HPV infection) as in other studies [14,22], but type 18 was less common (9%) than other high-risk HPV types.

In the TRICIN study, patients with HPV-negative CIN were excluded from the study; all patients in the Valencia et al. study had HPV. In our study, HPV-negative CIN was not an exclusion criterion, and 20% of patients were HPV-negative at enrollment. In another Spanish study, 26.8% of patients with an abnormal cytology smear were also HPV negative, but in cases with CIN 2/3 lesions, 12.2% of patients were HPV negative [23]. A negative HPV test in patients with CIN lesions might be interpreted as in the regressing stage or false-negative. The nature of false-negative tests has been studied by Eltoum et al. They found that 50% of patients with a negative HPV result in cervical smear later found positive HPV in cervical biopsy [24].

The study’s strength was that it was carried out in the colposcopy center by a certified colposcopy specialist. Results were evaluated not only by cytological examination or HPV testing but also by histological testing of biopsies. This study also has its limitations—it was an open-label, single-center study.

## 5. Conclusions

In conclusion, the vaginal spray of glycyrrhizinic acid used 20 days per month for six months appears to decrease the risk of the progression of CIN 1 lesions and the need to perform LLETZ. Still, a similar effect is not observed after stopping the usage.

## Figures and Tables

**Table 1:** The baseline characteristics in both groups.

Characteristics	Total, *n* = 100	Epigen Group, *n* = 50 (Epigen 10-Day *n* = 19; Epigen 20-Day *n* = 31)	Control Group, *n* = 50	*p*-Value
Age, years ± SD	32.4 ± 7.6	32.5 (33.2; 32.0) ± 7.5	32.3 ± 7.8	0.886
Education basic secondary higher	6 (6%) 43 (43%) 51 (51%)	2 (4%) (0; 2) 16 (32%) (7; 9) 32 (64%) (12; 20)	4 (8%) 27 (54%) 19 (38%)	0.036
Age at the first intercourse	17.7 ± 2.1	17.8 (17.5; 18) ± 2.4	17.7 ± 1.8	0.704
Number of lifetime partners	5 ± 3.2	5 (4.6; 5.3) ± 3.4	5 ± 3.1	0.951
Number and proportion of women who had 6 partners or more during their lifetime	30 (30%)	17 (34%) (5; 12)	13 (26%)	0.383
Duration of the partnership with the current partner, months	64 ± 73.4	66.4 (82.4; 56.5) ± 71.0	61.6 ± 76.4	0.749
Smoking	30 (30%)	15 (30%) (5; 10)	15 (30%)	1
Women who had any type of HPV at inclusion	80 (80%)	39 (78%) (15; 24)	41 (82%)	0.617
Women who were HPV-negative at inclusion	20 (20%)	11 (22%) (4; 7)	9 (18%)	0.683
Women who had high-risk HPV at inclusion	78 (78%)	39 (78%) (15; 24)	39 (78%)	1
Women who had only low-risk HPV at inclusion	2 (2%)	0	2 (4%)	0.495
Contraception				0.192
none	30 (30%)	14 (28%) (7; 7)	16 (32%)	
coitus interruptus	31 (31%)	20 (40%) (9; 11)	11 (22%)	
calendar method	1 (1%)	0	1 (2%)	
condoms	20 (20%)	7 (14%) (5; 9)	13 (26%)	
oral contraceptive	11 (11%)	7 (14%) (0; 7)	4 (8%)	
vaginal ring, contraceptive patch	6 (6%)	2 (4%) (1; 1)	2 (4%)	
intrauterine system	1 (1%)	0	1 (2%)	
intrauterine device	2 (2%)	2 (4%) (2; 0)	0	
other	3 (3%)	1 (2%) (0; 1)	2 (4%)	

SD—standard deviation; HPV—human papillomavirus.

**Table 2:** Epigen 10-day and control group results at the 1st and 2nd follow-up visit. Intention-to-treat analysis and per-protocol analysis.

	Intention-to-Treat Analysis			Per-Protocol Analysis		
Variables	Epigen 10-Day, Improvement/Constant/Deterioration, %	Control Group, Improvement/Constant/Deterioration, %	*p*-Value	Epigen 10-Day, Improvement/Constant/Deterioration, %	Control Group, Improvement/Constant/Deterioration, %	*p*-Value
	1st follow-up visit					
	*n* = 19	*n* = 50		*n* = 17	*n* = 47	
Cytological changes	47.4/36.8/15.8	44/46/10	0.7	47.1/35.3/17.6	46.8/42.6/10.6	0.79
Colposcopic changes	21.1/73.3/5.3	14/70/16	0.526	23.5/70.6/5.9	14.9/68.1/17.0	0.515
Histological changes	11.8/58.8/29.4	6.7/62.2/31.1	0.819	12.5/56.3/31.3	7.1/59.5/33.3	0.813
	2nd follow-up visit					
	*n* = 15	*n* = 35		*n* = 13	*n* = 32	
Cytological changes	46.7/46.7/6.7	51.4/45.7/2.9	0.882	46.2/46.2/7.7	56.3/40.6/3.1	0.644
Colposcopic changes	20/80/0	40/48.6/11.4	0.112	23.1/76.9/0	43.8/43.8/12.5	0.182
Histological changes	0/78.6/21.4	23.5/55.9/20.6	0.129	0/75/25	25.8/51.6/22.6	0.143

SD—standard deviation; HPV—human papillomavirus.

## Data Availability

All data were incorporated into the article.
